# Ketogenic diets as an adjuvant therapy for glioblastoma (KEATING): a randomized, mixed methods, feasibility study

**DOI:** 10.1007/s11060-020-03417-8

**Published:** 2020-02-08

**Authors:** Kirsty J. Martin-McGill, Anthony G. Marson, Catrin Tudur Smith, Bridget Young, Samantha J. Mills, M. Gemma Cherry, Michael D. Jenkinson

**Affiliations:** 1 Institute of Translational Medicine, University of Liverpool, Brownlow Hill, Liverpool, L69 3BX UK; 2 Department of Clinical Sciences and Nutrition, University of Chester, Chester, UK; 3 The Walton Centre NHS Foundation Trust, Lower Lane, Liverpool, L9 7LJ UK; 4 Department of Psychological Sciences, University of Liverpool, Brownlow Hill, Liverpool, L69 3BX UK; 5 Clinical Health Psychology Service, Royal Liverpool University Hospital, Liverpool, L7 8XP UK; 6 Department of Health Services Research, University of Liverpool, Brownlow Hill, Liverpool, L69 3BX UK; 7 Department of Biostatistics, University of Liverpool, Brownlow Hill, Liverpool, L69 3BX UK

**Keywords:** Glioblastoma, Ketogenic diet, Feasibility, Mixed-method, Pilot

## Abstract

**Purpose:**

We conducted a feasibility study to investigate the use of ketogenic diets (KDs) as an adjuvant therapy for patients with glioblastoma (GBM), investigating (i) trial feasibility; (ii) potential impacts of the trial on patients’ quality of life and health; (iii) patients’ perspectives of their decision-making when invited to participate in the trial and (iv) recommending improvements to optimize future phase III trials.

**Methods:**

A single-center, prospective, randomized, pilot study (KEATING), with an embedded qualitative design. Twelve newly diagnosed patients with GBM were randomized 1:1 to modified ketogenic diet (MKD) or medium chain triglyceride ketogenic diet (MCTKD). Primary outcome was retention at three months. Semi-structured interviews were conducted with a purposive sample of patients and caregivers (n = 15). Descriptive statistics were used for quantitative outcomes and qualitative data were analyzed thematically aided by NVivo.

**Results:**

KEATING achieved recruitment targets, but the recruitment rate was low (28.6%). Retention was poor; only four of 12 patients completed the three-month diet (MCTKD n = 3; MKD n = 1). Participants’ decisions were intuitive and emotional; caregivers supported diet implementation and influenced the patients’ decision to participate. Those who declined made a deliberative and considered decision factoring diet burden and quality of life. A three-month diet was undesirable to patients who declined and withdrew.

**Conclusion:**

Recruitment to a KD trial for patients with GBM is possible. A six-week intervention period is proposed for a phase III trial. The role of caregivers should not be underestimated. Future trials should optimize and adequately support the decision-making of patients.

**Electronic supplementary material:**

The online version of this article (10.1007/s11060-020-03417-8) contains supplementary material, which is available to authorized users.

## Introduction

Glioblastoma (GBM) is the commonest malignant primary brain tumor in adults, affecting 2–3 per 100,000 per year [1]. Even with maximal safe resection, radiotherapy and temozolomide [2, 3], the prognosis remains poor [4]. In the last 10 years, a series of large-scale clinical trials testing targeted therapies have all reported negative results [4–6] with no change in the current standard of care. Patients and caregivers often explore other alternative treatment options, including dietary changes such as the ketogenic diet (KD). Indeed, the James Lind Alliance Priority Setting Partnership identified that ‘the effect of lifestyle factors, including diet, on tumor growth’ to be a top 10 research priority for the neuro-oncology community [5].

KD is an ‘umbrella term’ used to describe high fat, low carbohydrate, adequate protein diets which promote the utilization of fat for energy, in the form of ketones. Initially hypothesized to work by exploiting the ‘Warburg effect’ [6–9], newer theories have proposed other mechanisms of action, with ketone bodies and medium chain triglyceride (MCT) fats playing a role in tumor metabolism, rather than or in addition to a reduction in glucose [10–13]. Animal models of glioma have shown that KD potentiates the effects of radiotherapy [14], reduce peri-tumoral edema [15] and reduce tumor angiogenesis [7]. In patients with gliomas, the evidence for KD is limited to case studies and single case reports [16–22]; all utilizing different KDs at different time points in the treatment pathway. No studies have been powered to assess efficacy.

Prior to designing and undertaking an adequately powered randomized control trial (RCT) investigating the efficacy of KDs in the therapeutic management of GBM, feasibility must be demonstrated [23–25]. We conducted the KEATING study to i) investigate protocol feasibility; ii) explore the potential impact of the study on patients’ quality of life and health; iii) explore patients’ perspectives of their decision-making when invited to participate in the study; and iv) optimize future phase III clinical trial design, whilst comparing two different KDs in an NHS setting.

## Methods

### Study design

KEATING consisted of two parts; a pilot study and a qualitative study. To investigate the feasibility of KDs as an adjuvant therapy for patients with newly diagnosed GBM undergoing chemoradiotherapy a prospective, non-blinded, single-center, randomized pilot study was undertaken. Twelve patients were randomized in a 1:1 ration to either medium chain triglyceride ketogenic diet (MCTKD) or modified ketogenic diet (MKD). A three-month dietary intervention was planned (primary end point), following which patients could choose to continue with the diet for a total of 12 months (secondary end point). To explore the decision-making of patients’ invited to participate in KEATING a qualitative study was embedded, interviewing patients who participated and declined, along with their caregivers. Ethical approval was granted by North West-Greater Manchester West Research Ethics Committee (17/NW/0013). KEATING was registered with the International Standard Randomized Controlled Trial Number Registry (reference number 71665562) and ClinicalTrials.gov (reference number NCT03075514). The KEATING pilot study protocol has been published previously [26] (substantial amendments are detailed in the online resources).

### Participants

Patients were recruited from a single adult neuroscience center. Patients were eligible if they were ≥ 16 years, ECOG performance status 0–2, had histologic diagnosis of GBM (WHO grade IV [27] within last four months (biopsy of surgical resection), were planned to undergo radiotherapy and temozolomide chemotherapy [2]. Patients were not eligible if they had any prior use of a KD, kidney, liver or gallbladder dysfunction, Metabolic or eating disorder, Body mass index (BMI) ≤ 18.5 kg/m^2^, were taking weight loss medications, pregnant or breastfeeding, or had Medical conditions that may increase risks associated with KD.

### Randomization

Patients were randomized to either MCT KD or MKD using 'sealedenvelope'™ randomization system and a permuted block randomization method, ensuring similar numbers in each group, at a ratio of 1:1. This was constructed and administered by the study statistician (CTS), who was not involved with recruiting patients, thus concealing the sequence of allocation. Patients were then informed of their dietary intervention group by telephone and initiated diet within five working days of consent.

### Dietary intervention and procedures

Two KDs were included in KEATING; MCTKD and MKD. A comparison of the macronutrient content, example meal plan and monitoring requirements for each diet can be found in Online Resource 1, Table A. Patients and their caregiver (if present) received dietary education from the dietician and were provided with a bespoke seven-day meal plan, recipes, dietary information sheets and food diaries. MCT was provided as Betaquik® (Vitaflo International Ltd), a nutritional product available by prescription. Patients were reviewed by telephone at weeks one, three and nine, and in an outpatient’s clinic at weeks six and twelve. Patients who wished to continue with the diet were then reviewed at six, nine and 12 months. Urinary ketones were monitored twice daily for the first six weeks, then weekly thereafter using Ketostix® (Bayer, Germany). Blood ketones and glucose levels were monitored once weekly using GlucoMen Aero 2 K® home monitoring kit (Abbott Laboratories, UK). All surgical and oncological interventions were undertaken as per current standard of care [28].

### Outcomes

The primary outcome for KEATING was to estimate retention rate at three months to inform sample size calculations for future definitive trials. Secondary outcomes included estimations of recruitment rates, enrolment rates, long term retention rates and to obtain data on dietary compliance through food diaries, ketosis through ketone diaries, dietetic time to complete intervention, protocol refinements, completeness of data, quality of life assessed using EORTC QLQ C30 and BN20 questionnaires, food acceptability assessed through questionnaire, gastrointestinal side effects graded using Common Terminology Criteria for Adverse Event (CTCAE) reporting, biochemical markers (renal, bone, liver function tests, fasting lipid and fasting glucose) and anthropometry (weight, BMI, hand grip strength, mid arm muscle circumference, free fat mass and waist circumference. All outcomes were assessed at three months and twelve months.

Pilot success for KEATING was graded using a predetermined traffic light system (≥ 75% to proceed, ≥ 50% required review and < 50% study closure), which considered recruitment success, retention rates, dietary acceptability, the commencement of diet pre chemoradiotherapy and extent of missing data [29]. Magnetic Resonance Imaging (MRI) was used to interpret tumor progression. Progression free survival (PFS) was defined as the time from date of surgery randomization to date of recurrence on MRI. Recurrence was defined by a Neuroradiologist using the Response Assessment in Neuro-Oncology (RANO) criteria [30]. Overall survival (OS) was defined as the time from date of surgery to date of death from any cause. Adverse events (AE) and serious adverse events (SAE) were also reported as per CONSORT guidelines for pilot and feasibility studies [31].

### Statistical analysis

Previous feasibility work estimated recruitment targets of one patient per month [32], in keeping with National Institute for Health Research (NIHR) funded trials [33]. Twelve patients were to be recruited over 12 months. Descriptive statistical methods were used and PFS and OS were PFS and OS were assessed Kaplan–Meier survival curves. During the course of the pilot study it was clear that retention on diet was an issue and with agreement from the Trial Steering Committee, a sub analyses was introduced at week six, with a view to providing further information which would inform the design of later trials. This was not included in the original study protocol (for amendments see Online Resource 2).

### Qualitative study

Due to poor recruitment in the early stages of the KEATING study, we proceeded to amend the protocol to embed a qualitative component to explore patients’ decision-making about KEATING. Participants for the qualitative study were a purposively sampled sub-set of patients and their caregivers, who had been approached to participate in KEATING [34, 35]. Sampling was informed by the review of screening logs maintained as part of KEATING and aimed to include both those who consented and declined, those randomized to MCTKD and those to MKD. Patients were interviewed retrospectively, up to three months after being approached about KEATING. Adequate sample size was determined using the ‘information power’ concept [36, 37]. The interviews were conversational, patient-centered, topic guided (see Online Resource 3, Table A) and iterative. The topic guide was devised by two members of the research team (KM, GC). The researcher conducting the interviews had a dual role (dietician and qualitative researcher), therefore, interviews were reflexive and conducted in a gentle, sensitive and non-judging manner, to make the experience as comfortable as possible for patients. Interviews were audio-recorded and transcribed. Analysis drew on the Braun and Clarke thematic approach to identify patterns of meaning within the data [38]. KM lead a process of iterating between the developing analysis and new data (familiarization). Other members of the qualitative study team (BY and GC) read a sub-set of transcripts and developed the analysis by periodic discussion. Integration between KEATING and the embedded qualitative study took place after individual analysis had occurred. Integration was conducted by three authors (KM, GC, BY).

## Results

### KEATING participant characteristics

**Fig. 1:**
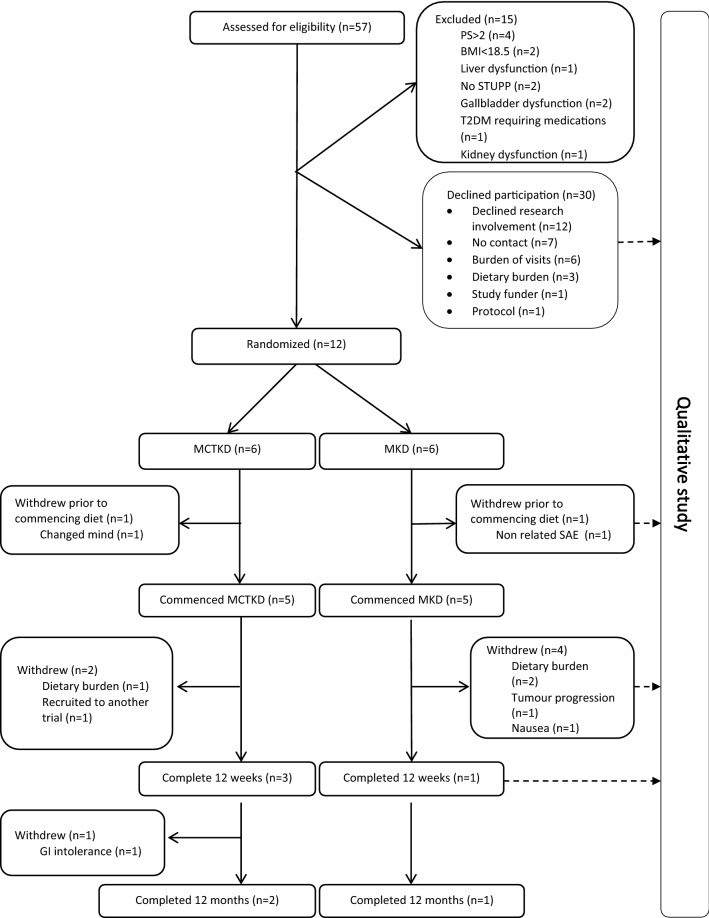
CONSORT diagram for KEATING

Between 1st April 2017 and 8th February 2018 we assessed 57 patients for eligibility. Fifteen were ineligible (26.3%), 30 declined (52.6%) and 12 (21.1%) were randomized. Of those recruited eight were male and four female, with a median age of 57 years (44–66 years). Figure 1 shows the patient flow through the study and Table 1 presents the demographic and clinical characteristics of patients who were randomized.

**Table 1:** Patient demographics and clinical characteristics

Trial N^o^	Gender	Age (yrs)	Tumor location	Treatment	Pathology			DEX (mg/d) (median [range])	Study arm	Duration on diet (weeks)	PFS (weeks)	OS (weeks)
					*MGMT*	*IDH-1*	*ATRX*					
T01	Male	53	Right temporal	GTR, RT, TMZ	Unmethylated	Wildtype	Retained	5 (2–4)	MCTKD	22.4	32.4	35.4
T13	Male	49	Left parietal	NTR, RTX, TMZ	Unmethylated	Wildtype	Retained	4 (0)	MCTKD	5.1	14.4	60.6
T23	Female	54	Left frontal	NTR, RTX, TMZ	Unmethylated	Wildtype	Retained	4 (0)	MCTKD	5.7	44.4	83.6
T27	Female	62	Right occipital	GTR, RTX, TMZ^b^	Methylated	Wildtype	Retained	2 (0)	MKD	0	5.1	NR^e^
T28	Male	64	Left temporal	Bx, RTX, TMZ^a^	Unmethylated	Wildtype	Retained	4 (3–4)	MKD	7	13.1	67.3
T39	Female	66	Right parietal	NTR, RTX, TMZ	Methylated	Wildtype	Retained	4 (0)	MKD	5.3	64.3	NR^e^
T44	Male	44	Right temporal	GTR, RTX, TMZ	Methylated	Mutant	Mutated	NA	MKD	52	NA^d^	NR^e^
T45	Male	46	Left frontal	NTR, RTX, TMZ, Lomustine	Unmethylated	Wildtype	Retained	3 (2–3)	MCTKD	52	14.0	NR^e^
T47	Female	58	Right frontal	NTR, RTX, TMZ^a^	Inconclusive^c^	Wildtype	Retained	2 (0)	MKD	4.6	14.0	31.6
T51	Male	57	Left frontal	STR, RTX, TMZ	Methylated	Mutant	Mutated	1 (1–1.5)	MCTKD	52	NA^d^	NR^e^
T52	Male	60	Left frontal	NTR, RTX, TMZ^a^	Unmethylated	Wildtype	Retained	2 (0)	MCTKD	0	23.9	NR^e^
T57	Male	57	Right multifocal	Bx, RTX, TMZ^a^	Unmethylated	Wildtype	Retained	2 (0)	MKD	6	14.0	57.1

*ATRX* alpha thalassemia/mental retardation syndrome X linked, *Bx* biopsy, *DEX* dexamethasone, *GTR* gross total resection, *MCT KD* medium chain triglyceride ketogenic diet, *MGMT* O^6^-methylguanine-DNA methyltransferase, *MKD* modified ketogenic diet, *NA* not applicable, *ND* no data recorded by patient, *NR* not reached, *NTR* near total resection, *OS* overall survival, *PFS* progression free survival, *RTX* radiotherapy, *SD* standard deviation, *STR* subtotal resection, *TMZ* temozolomide, *Treatment* treatment received whilst following a ketogenic diet

^a^Unknown if completed full course of radiotherapy and chemotherapy as withdrew from study

^b^6 days of temozolomide not given

^c^Insufficient tissue to perform *MGMT* analysis

^d^No progression at time of reporting (08/MAR/2019)

^e^Alive at time of reporting (08/MAR/2019)

### Primary outcome: retention at three months

Of the 12 patients randomized in KEATING (n = 6 MCTKD; n = 6 MKD), two withdrew prior to commencing the diet (n = 1 MCTKD; n = 1 MKD). Reasons for withdrawal were non-dietary related SAE (n = 1) and patient change of mind (n = 1). Of the 10 patients who commenced diet, six withdrew before reaching the three month primary end point (n = 2 MCTKD; n = 4 MKD). The median duration until discontinuing the MCTKD was 38 days (36–40 days; n = 2) and for MKD was 39.5 days (32–49 days; n = 4).

### Secondary outcomes: protocol feasibility

#### Recruitment

Twelve patients were recruited over 12 months from a sample of 42 eligible patients, achieving a recruitment rate of 28.6% (or 21% of the overall screened population).

#### Long term retention

Of the 12 patients randomized in KEATING, four continued with their KD after month three (n = 3 MCTKD; n = 1 MKD). One patient (MCTKD group) then stopped at month six due to gastrointestinal side effects. In total, three patients completed the 12 month intervention period (n = 2 MCTKD; n = 1 MKD). These patients all chose to continue with their KD after completing the study.

#### Level of ketosis

During the first six weeks, 79.7% of MCTKD (n = 3) and 79.3% of MKD (N = 3) recordings were within the desired level of ≥ 4 mmol/L. Those who withdrew from the study reported lower urinary and serum ketone levels than those who stayed on diet up to month 12. The median level of urinary ketosis for each patient for their duration on diet is shown in online resource 3, figure A.

### Secondary outcomes: impact of the study on patients’ health

#### Quality of life

At baseline, there was little difference between the Global Health Status (GHS) of those patients who went on to withdraw and those who continued with their diet and were retained within the study, in either dietary group (withdrew MCTKD 72.2 ± 20.7 [n = 3]; retained MCTKD 75 ± 6.8 [n = 3]; withdrew MKD 70 ± 13.8 [n = 5]; retained MKD 80 ± 0 [n = 1]).

The GHS of those who withdrew from the study at week six, fell below the brain cancer reference value in both the MCTKD and MKD groups (withdrew MCTKD 41.7 ± 0 [n = 1]; withdrew MKD 50 ± 0 [n = 2]). For those who continued with their diet and were retained within KEATING, GHS improved for the patient following MKD and reduced for those patients following MCTKD. In both groups the GHS remained above the brain cancer reference value (retained MCTKD week six 66.7 ± 0 [n = 3]; retained MCTKD month three 66.7 ± 13.6 [n = 3]; retained MCTKD month 12 66.7 ± 8.4 [n = 2]; retained MKD 100 ± 0 [n = 1] from week six onwards) (see online resource 3, figure B).

#### Food acceptability

Food acceptability reduced from baseline in both groups. The lowest food acceptability scores were recorded at week six of following the diet (baseline MCTKD 60.7 ± 10.5 [n = 6]; baseline MKD 54.3 ± 6.2 [n = 6]; week six MCTKD 42 ± 8.9 [n = 4]; week six MKD 43.5 ± 12.8 [n = 4]). Food acceptability then improved between week six and three months (MCTKD 49 ± 2.9 [n = 3]; MKD 58 [n = 1]), but reduced slightly before the end of the study (MCTKD 47.5 ± 6.5 [n = 2]; MKD 53 [n = 1]).

#### Adverse and serious adverse events

There were five adverse events and three serious adverse events. Adverse events were due to hypokalemia (n = 2, CTCAE grade 1), hypernatremia (n = 1, CTCAE grade 1), hypocalcaemia (n = 1, not classified as adjusted calcium > 2 mmol/L) and a partial seizure (n = 1, CTCAE 1). Serious adverse events were due to post-operative wound infection (n = 1, CTCAE grade 3, resulting in withdrawal from the assigned dietary intervention), seizure (n = 1, CTCAE grade 2) and back pain (n = 1, CTCAE grade 2), none of which were related to the dietary intervention.

#### Survival analysis

**Fig. 2:**
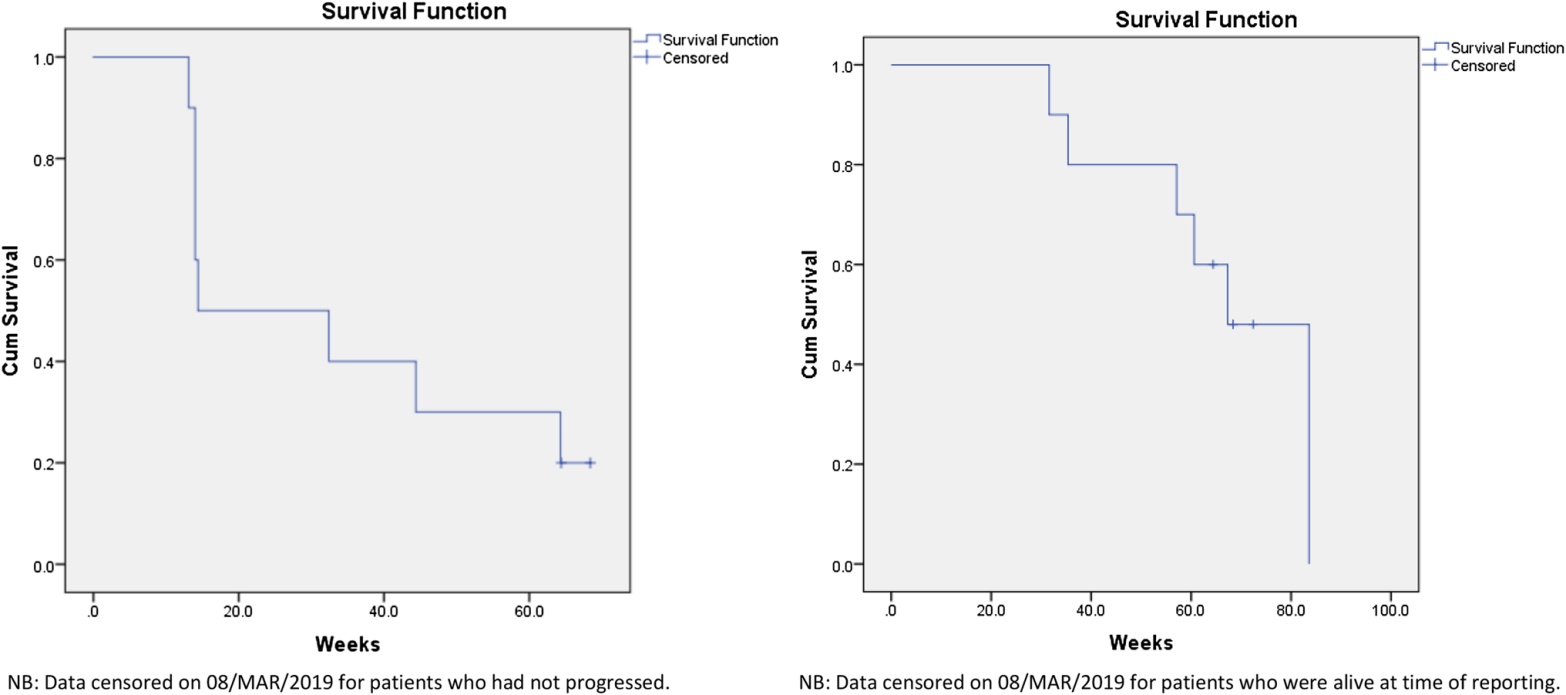
Progression free and overall survival of patients who commenced diet (MCT KD n = 5; MKD n = 5)

The median time to progression was 14.4 weeks (SE 14.6; 95% CI 0–42.9 weeks). Median overall survival was 67.3 weeks (SE 6.2; 95% CI 55–79.6 weeks). Survival analysis is illustrated in Fig. 2.

#### Additional outcomes

Additional outcome reporting as per the KEATING protocol can be located in online resource 3 for the following outcomes: enrolment of participants prior to, during and post chemoradiotherapy commencement; dietary compliance; dietary adjustments required to achieve ketosis; dietetic time required for dietary interventions; gastrointestinal side effects; changes to biomarkers; anthropometric changes; and determining pilot success.

### Qualitative study

#### Participant characteristics

Fifteen patients and their caregivers were invited to be interviewed. Between January and April 2018, 10 patients and five of their caregivers, all of whom were white British, were interviewed (Table 2). All participants were interviewed separately except one patient and one relative who were interviewed jointly. Individual interviews lasted for an average (median) of 44 min (36–62 min) and the dyad interview lasted 65 min.

**Table 2:** Patient and caregivers’ characteristics for those who participated in the qualitative study

KEATING participant number	Gender	Age (years)	IMD	KEATING intervention arm	KEATING categorization	Relative interviewed	Relative participant number	Gender	Relationship to participant
T27	Female	60–69	1*	MKD	Early withdrawal	No	–	–	–
T30	Female	70–79	> 50%^Ɨ^	–	Declined	Yes	T30/R	Male	Husband
T35	Female	50–59	4*	–	Declined	No	–	–	–
T39	Female	60–69	30–50%^Ɨ^	MKD	Delayed withdrawal	Yes	T39/R	Male	Husband
T44	Male	40–49	2*	MKD	Continued participation	No	–	–	–
T45	Male	40–49	7*	MCTKD	Continued participation	Yes	T45/R	Female	Wife
T47	Female	60–69	2*	MKD	Delayed withdrawal	Yes	T47/R	Male	Husband
T51	Male	50–59	10*	MCTKD	Continued participation	Yes	T51/R	Female	Wife
T52	Male	60–69	2*	MCTKD	Early withdrawal	No	–	–	–
T55	Male	60–69	8*	–	Declined	No	–	–	–

Key: Continued participation = continued with the intervention beyond three months; Early withdrawal = withdrew from KEATING following consent and randomization, but prior to commencing a KD; Delayed withdrawal = withdrew from KEATING after commencing KD but before the primary end point of three months; Declined = declined to participate in KEATING. Time to interview = time from initial contact about KEATING to qualitative interview

*IMD* index of multiple deprivation, *MCT KD* medium chain triglyceride ketogenic diet. *MKD* modified ketogenic diet

*Index of Multiple Deprivation (England): decile of 1 = 10% most deprived areas of England, decile of 10 = 10% least deprived areas of England; ^Ɨ^Index of Multiple Deprivation (Wales): 10% = 10% most deprived areas of Wales, > 50% =  > 50% least deprived areas of Wales

#### Integrated results of KEATING and the embedded qualitative study

Table 3 integrates the findings of the KEATING and the embedded qualitative study, using an adapted triangulation protocol [39]. Throughout the table, we present patients’ verbatim quotes in speech marks, with ellipses indicating missing text and square brackets indicating replacement or explanatory text. To preserve anonymity, all patients are identified by the patient’s KEATING study screening log number (e.g. T01) and caregivers by an associated number (e.g. T01/R). We highlighted if the results of the two studies converge, are complementary, are contradiction, or are silent [39]. Further detailed analysis of the qualitative study can be found via online resource 4.

**Table 3:** Integrated findings of KEATING and the embedded qualitative study

Theme	KEATING pilot study	Qualitative study	Convergence, complementary, contradiction, silence
1. Recruitment	Recruitment rate of 28.6%	For those patients who participated in KEATING their decision was intuitive and emotional: *“I jumped in, you know, took the opportunity with both hands … it was a no brainer”* (T44) and *“more of a gut decision”* (T52)*.* Participating offered them the opportunity to *“take control”* and *“fight for their life”* (T44) For those who declined, the decision was deliberative and considered, consistently describing a lack of perceived personal benefit from participation: *“the only thing I think about this study is what would benefit me”* (T35). One viewed KEATING as *“a waste of your life”* (T35)	Complementary. Findings from the qualitative research explain why some patients participated in KEATING, whilst others declined
		Both groups validated their decision, seeking approval from their caregiver: *“it was a case of speaking to my family and getting their support to make sure that they were on board with what I was going to do, my family gave me the thumbs [up]”* (T44) and some patients spoke of discussing their decision to participate with their relative, sharing the decision: *“we had a discussion together as to whether or not we felt it was the right thing for me to do… [caregiver] just supported me with it, he felt that I should be giving it a go as well”* (T47)	
2. Retention	33% retention rate at 3 months (MCT KD n = 3; MKD n = 1)	Those who continued to participate in KEATING spoke positively about the diet and related retention to support from their caregiver. Caregivers were supportive and emphasized the diet to be “*a new normal for us”* (T45/R)	Complementary. Findings from the qualitative research identifies why some patients withdrew from KEATING. The qualitative study also identifies patients’ motivations for continuing to participate in KEATING
	25% retention rate at 12 months (MCT KD n = 2; MKD n = 1)	Patients validated their decision to continue on a regular basis making reference to the influence of ‘positive stories’ from long term ketogenic-glioblastoma survivors: *“there's lot of good results of people having positive responses to it [ketogenic diet]… the one story was the guy who had a, erm had the same tumor, he’s on this [ketogenic diet], his [tumor] reduced, what's not to want to go for that?”* (T45). They also found motivation from external sources such as *“clear scans”* (T44), with ketones providing *“a quick confidence check and every now and again”* (T45)	
	Median duration until discontinuing the MCT KD was 38 days (36–40 days; n = 2) and for MKD was 39.5 days (32–49 days; n = 4)	Those who withdrew, spoke of negative experiences which reduced their quality of life: *“I was worrying, I was waking up, I was literally waking up… and that’s all I could think about: ‘Oh I've got to get my fats intake today’. And it was pulling me down”* (T39). They also reported finding low ketones *‘demoralizing’*	
3. Role of caregivers	No data	The caregivers of those patients who participated in KEATING also described their decision as instantaneous, “*I’d take anything with open arms because anything that would help cure [the tumor], you know… I’d jump at it”* (T47/R), with caregivers attributing a kind of selfishness to their motives: “*I wanted her to have a go… I suppose it’s a bit selfish really but you know you, there’s a selfish element in it because you want her to be here sort of thing”* (T39/R)	Silence in KEATING, whilst the qualitative study offered insight into the role of the caregiver in the decision-making process and in supporting the patient to implement the intervention
		For patients who declined, caregivers generally agreed with the patients’ decision in relation to quality of life: *“well I think it’s something to be worthwhile but, erm, I was a bit concerned that it was a very restrictive diet for my wife to take at this stage really”* (T35/R)	
		In relation to retention, patients also reported caregivers to have an important role. Those who participated in KEATING required support both practically and emotionally, with caregivers emphasizing the diet to be “*a new normal for us”* (T45/R). Whilst those who withdrew sought their relative opinion and support in their decision to withdraw: “*it’s too long on the diet*” (T47/R)	
4. Quality of life	Reduced from baseline	Those who initially consented to participate in KEATING and later withdrew reported the diet to have a negative impact on their quality of life: *“I was worrying, I was waking up, I was literally waking up… and that’s all I could think about: ‘Oh I've got to get my fats intake today’. And it was pulling me down”* (T39)	Complementary. The qualitative study offered further information about patients’ perceptions of the importance of quality of life, over the course of the study, and how this impacted their decision to retain or withdraw
		Whilst those who continued to participated reported the diet to offer “*a great quality of life with cancer”* (T44)	
		Those who declined to participate considered the impact of the diet on their quality of life as part of their decision-making: *“You get to around 70 years old and that’s where I am. So now every day I get up I want a quality day…and so having a complex regime around diet again it doesn’t appeal”* (T55), with one viewing the KEATING as *“a waste of your life”* (T35)	
5. Dietary acceptability	Reduced from baseline	For those who declined to participate and those with withdrew, a three month dietary intervention was considered to be *‘too long’* and unsustainable to *“live with that forever more”* (T47), but reflected that they might have considered participating in KEATING for *“half of the time”* (T35)	Complementary. Dietary acceptability reduced from baseline in all but one patient (MKD). The qualitative study enhanced researcher understanding of a realistic and acceptable timeframe for the dietary intervention

*Convergence* uniformity within the quantitative and qualitative findings, *Complementary* quantitative and qualitative results enhance the qualities of each other, *Contradiction* quantitative and qualitative results oppose each other, *Silence* no data or results to compare to the opposing research method

## Discussion

This randomized, pilot study with an embedded qualitative design was designed to explore the feasibility of KD trials for patients with glioblastoma, with a view to recommending improvements to optimize the design of future phase III trials.

KEATING recruited to time and target, despite an initial slow start. The recruitment rate (28.6% of the eligible population) was much lower than NIHR HTA funded oncology clinical trials (50 to 89%) [33], but in keeping with recent survey data from the National Brain Tumour Society, with 21% of patients with brain tumors participating in clinical trials [40, 41]. Screening log data at the start of KEATING revealed that patients were declining to participate due to (i) not wanting to participate in research; (ii) the burden of dietitian visits; and (iii) the burden of KD. During the qualitative study those patients who declined to participate in KEATING identified their quality of life as an important factor in decision-making, and this aspect was not detected in the screening log data or in previous surveys regarding participation barriers [40]. These patients also spoke of the role of caregivers in influencing their decision to participate or not in KEATING, an aspect highlighted in trials elsewhere [41]. In contrast, patients who consented to KEATING made an intuitive and emotional decision, later reflecting that this decision was based upon quantity rather than quality of life. This optimism surrounding longevity of life is often used as a coping strategy by patients and may not invalidate their informed consent. There is currently little guidance offered by the Health Research Authority (HRA) Good Clinical Practice guidelines [42] regarding this matter, thus clinicians should continue to use their clinical judgement when assessing patients’ informed consent to participate in trials. Our findings are in keeping with recent publications highlighting the need for improved recruitment strategies and decisional support in neuro-oncology populations [40, 41].

The retention rate in KEATING was lower than anticipated. Out of the 12 patients randomized, 10 commenced KD and only four met the primary endpoint of three-month dietary intervention (1 KD, 3 MKD). Cancer trials in general report median retention rates of 89% (IQR 79–97% with valid primary outcome data at follow up) [33] and previous KD studies for patients with GBM report retention rates of 50 to 100%, with retention determined at eight weeks [19], three months [18, 32] and the point of tumor progression [17]. However, patients in these previous KD studies self-selected to try the diet, mainly at recurrence or post-treatment, creating an optimistic bias in retention, when compared to the general unselected GBM population approached for KEATING. Those who withdrew from KEATING did so either after randomization but prior to commencing the diet (n = 2) or after following the KD for approximately six weeks (MCTKD median 38 days [36 to 40 days], n = 2); MKD 39.5 days [32 to 49 days], n = 4), during which time patients were undergoing radiotherapy and concomitant temozolomide chemotherapy. Patients reported their reasons for withdrawal to be related to dietary burden and side effects, in particular nausea, which could have been related to the chemotherapy. Those continued on their assigned KD were generally younger patients with more favorable prognostic features (*MGMT* methylated; *IDH-1* mutant) and this may also have influenced their ability to stay on the study and implement the diet.

The reasons for poor retention on diet were explored in our qualitative study. Those who withdrew spoke of finding their low urinary and blood ketones to be ‘demoralizing’, feeling that the diet was not working, and withdrawing due to the negative effect this feeling had on their quality of life. This was confirmed in the quality of life data as patients who withdrew reported their global health status (GHS) to be below the brain cancer reference value at week six of the study. We appreciate multiple factors can affect the quality of life for these patients and whilst ketones are used to monitor the diet, urinary ketones not always robust markers of compliance and can be effected by hydration levels and the use of dexamethasone. Therefore low ketones may demoralize patients, even when they appear to be following the diet robustly.

For those patients who continued to participate in the trial to 12 months, GHS reduced within the MCTKD group and improved in the MKD group. However, during the qualitative interviews, both groups reported to experiencing a ‘fantastic quality of life’ describing the diet as offering a sense of ‘control’ whilst receiving their tumor treatment. Although the EORTC QLQC30 and BN20 questionnaires are validated for patients with glioblastoma, they are time consuming to complete and some questions are not relevant for patients following KDs. It may be beneficial for future KD trials to reduce the length of the questionnaire and therefore patient burden, focusing particularly on GHS, as these questions provided the most insight in KEATING.

During interviews, patients reported several motivational factors for continuing with the diet, including through online blogs of long term glioblastoma survivors, positive MRI results and high ketone levels, using these as a means of validating their decision to stay on diet. This corroborates the findings from KEATING since high ketones indicted compliance with KD. Patients with higher ketones stayed in the study, whilst those with lower ketones withdrew early, at around week six. Furthermore, a pilot study for KD in patients with other advanced cancers (breast, ovarian, lung, gastrointestinal), also experienced similar retention rates to KEATING (retention rate 31%, n = 5 of 16), in a trial which permitted a more liberal KD (70 g of carbohydrates per day) and where all food provision was provided [43]. Thus, a more flexible dietary approach may not be the simple solution.

The high withdrawal rates in KEATING suggest that a three-month KD intervention may be too long for most patients. Our qualitative study also highlighted that those who withdrew considered the three-month intervention to be undesirable, an opinion also reflected by those who declined, further corroborating findings from KEATING. A shorter, six-week intervention, is likely to be more tolerable and acceptable to patients. This could be offered alongside radiotherapy and concomitant chemotherapy, which coincides with the proposed optimal time for the diet derived from animal model data [14]. Offering the diet at the same hospital site as the radiotherapy would aid a timely start of the diet. Whilst patients reported ‘feeling free again’ once the diet was discontinued, it is important to note, that this may not necessarily equate to dietary acceptability outside of a clinical trial. In a future post-trial environment, information regarding the efficacy of the diet may be available, which could alter patients’ willingness to engage.

The qualitative study also highlighted caregivers to be key in supporting patients to implement the diet. The role of caregivers, both in the decision-making of patients and the ongoing support offered, were aspects that were underappreciated in KEATING. A recent KD study for patients with Alzheimer’s disease, also highlighted dietary and caregiver burden to be influential over patient withdrawal [44].

KEATING had several limitations. The return rate of food and ketone diaries was low at 12 months, subsequently affecting the analysis. This is a common problem in dietary intervention trials, given the time commitment required to return diaries. All accounts of dietary intake were also self-reported and at risk of reporter bias. The sample size for the qualitative was small and we cannot be certain that saturation was achieved. Nevertheless, drawing on the concept of ‘information power’ in qualitative research [36], this study had a well specified aim, and it has provided insights that will be valuable in informing a future phase III trial. Some patients were also interviewed up to three months after their decision about KEATING. They may have found it difficult to accurately recall their decision-making process, particularly given the nature of their condition and the numerous other decisions they will likely have had to make regarding their care and treatment.

In order to optimize protocol feasibility and patient experience future trials should consider the following suggestions:

- To assess effectiveness in a phase III trial a six–week diet intervention period would be deliverable.
- To optimize recruitment and retention a longitudinal, prospective, qualitative study, which focuses on patient and caregivers understanding and decision-making in the context of trial participation should be embedded within KD trials.
- Future phase III trials would benefit from an internal pilot to further test the recommendations derived from KEATING, focusing on stop/ go criteria for staged recruitment, retention at 6-weeks and commencement of diet prior to chemoradiotherapy.

In conclusion, recruitment of patients with GBM to a KD trial is possible. To assess efficacy in a phase III clinical trial, a six-week intervention period is proposed. The role of caregivers in the patients’ decision-making process and in supporting patients to implement KDs should not be underestimated.

## Electronic supplementary material

Below is the link to the electronic supplementary material.

Supplementary file1 (DOCX 12 kb)

Supplementary file2 (DOCX 15 kb)

Supplementary file3 (DOCX 31 kb)

Supplementary file4 (DOCX 114 kb)

## Data Availability

The datasets generated and analysed during the current study are available at the University of Liverpool repository https://doi.org/10.17638/datacat.liverpool.ac.uk/692
