# Supplementary material for: Ketogenic diets as an adjuvant therapy for glioblastoma (KEATING): a randomized, mixed methods, feasibility study
Source: J Neurooncol. 2020 Feb 8;147(1):213–27. doi: 10.1007/s11060-020-03417-8 (PMC7076054; doi:10.1007/s11060-020-03417-8)
Supplement: Supplementary file 1 — Supplementary file1 (DOCX 12 kb) [file 11060_2020_3417_MOESM1_ESM.docx]

**Online resource 1: A comparison of MCTKD and MKD dietary interventions**

| **Supplementary table A: Comparison of MCTKD and MKD dietary interventions** | | |
| --- | --- | --- |
| **Intervention** | **MCTKD** | **MKD** |
| Macronutrients | **10%** carbohydrate  **75%** fat  (**30% of which from MCT nutritional products**)  **15%** protein | **5%** carbohydrate  **80%** fat  **15%** protein |
| Example meal | Bacon x 2, eggs x2 scrambled with **2** **dsp double cream**, ½ avocado**, 1 ½ tomatoes**, **½ slice low carb bread**, handful spinach and **100ml Betaquik MCT nutritional product** | Bacon x 2, eggs x2 scrambled with **4** **dsp double cream**, ½ avocado, handful spinach and **mushrooms fried in 1 tsp oil** |
| Requirements of patients | Urinary ketones twice daily  Blood glucose and ketones weekly  Intermittent food diaries  Dietitian appointments | Urinary ketones twice daily  Blood glucose and ketones weekly  Intermittent food diaries  Dietitian appointments |

Abbreviations: dsp= dessertspoons; LCT = long chain triglyceride; MCT = medium chain triglyceride; MCT KD = medium chain triglyceride ketogenic diet; MKD = modified ketogenic diet; tsp= tablespoon.
