# Supplementary material for: Ketogenic diets as an adjuvant therapy for glioblastoma (KEATING): a randomized, mixed methods, feasibility study
Source: J Neurooncol. 2020 Feb 8;147(1):213–27. doi: 10.1007/s11060-020-03417-8 (PMC7076054; doi:10.1007/s11060-020-03417-8)
Supplement: Supplementary file 2 — Supplementary file2 (DOCX 15 kb) [file 11060_2020_3417_MOESM2_ESM.docx]

**Online resource 2: Amendments to KEATING protocol**

1. *Amendments to protocol*

Further amendments were also undertaken, these included: i) expanding the inclusion criteria, with a view to broadening the study population, by including biopsy patients and those patients receiving radiotherapy or chemotherapy, rather than combined chemoradiotherapy only treatments; ii) extending the recruitment period by 12 months (24 month recruitment period in total); iii) patients permitted to commence KD post radiotherapy but within four months of initial surgery, with a view to promoting uptake.

1. *Overview of the qualitative study topic guide*

| **Supplementary table A: Overview of the qualitative study topic guide** | | |
| --- | --- | --- |
| Section | Example questions | Example prompts |
| Study information and recruitment process | Who initially told you about the study? | Can you tell me about the run up to the study? Can you remember when you were first diagnosed? Is there anything would you change about how you were approached? When would you prefer to be approached? Who would you prefer to approach you about the study? |
|  | What is your understanding of the reason why the study is being done? | What is your understanding of what the study aimed to investigate? Are you able to tell me a bit about what this involves for patients? |
|  | We posted an information sheet to you about the study (demonstration sheet provided). What are your views on it? | Did you find the wording and layout suitable? Is there anything that you would change? Were any parts helpful? Did you have any questions after reading the leaflet? |
| Decision-making | Before making your decision about taking part did you seek information from anywhere else? | Did you discuss your involvement in the study with your family or friends? Did you discuss the study with a doctor or nurse? |
|  | Can you tell me about how you came to your decision to take part/not take part in the study? | When did the decision start to form in your mind? How do you usually make decisions (gut reaction or evidence based/ informed decision)? How certain were you with your decision? How confident were you with your decision? |
|  | Are there any potential changes that could be made to alter your opinion? | Any changes to the study design? Changes to how you are told about the study and what it involves? |
|  | When thinking about the treatment you have received, is there any time when you think starting the diet would be most appropriate? | This could be not at all, before/after surgery, before/ during/ after radiotherapy or before/ during or after chemotherapy or after all treatment has finished. Can you tell me a bit more about that? |
|  | Did you consider any other studies or treatments? | Other studies that may be open to you locally or nationally? Have you taken part in research previously? Would you take part in potential future studies? Have you looked into any other diets or nutritional supplements? |
|  | If you had the chance to be involved in the study again would you make the same decision? | If changed decision why is that? |
| Experience of KEATING (KEATING participants only) | What did you think when you were told about your allocated diet? | Did you have any worries or expectations? Do you have any particular views on being randomized to one diet or the other? |
|  | What has your experience of the KEATING study been so far? | Has your experience influenced your views on research? Do you have any tips for a similar study in the future? Has any part been particularly burdensome? Have there been times when the diet has been more difficult to follow? Have there been times when the diet has been easier to follow? |
| Conclusion | Is there anything else you would like to talk about that we haven’t covered? Do you have any questions for me? Thank you for taking the time to talk to me. | Can you tell me more about that? What is your understanding of that? |
