# Supplementary material for: Ketogenic diets as an adjuvant therapy for glioblastoma (KEATING): a randomized, mixed methods, feasibility study
Source: J Neurooncol. 2020 Feb 8;147(1):213–27. doi: 10.1007/s11060-020-03417-8 (PMC7076054; doi:10.1007/s11060-020-03417-8)
Supplement: Supplementary file 3 — Supplementary file3 (DOCX 31 kb) [file 11060_2020_3417_MOESM3_ESM.docx]

**Online resource 3: KEATING additional tables, figures and outcome reporting**

1. Supplementary figure A: Median urinary ketone level for each patient for their duration on diet
2. Supplementary figure B: Global health status of patients who withdrew and retained in the trial

NB: At baseline withdrew MCT KD n=3, retained MCT KD n=3, withdrew MKD n=5, retained MKD KD n=3; at week six withdrew MCT KD n=1, retained MCT KD n=3, withdrew MKD n=2, retained MKD n=1; at month 3 retained MCT KD n=3, retained MKD n=1; at month 12 retained MCT KD n=2, retained MKD n=1.

1. *KEATING study secondary outcomes: Protocol feasibility*

Enrolment of participants prior to, during and post chemoradiotherapy commencement

All patients (n=12) were enrolled and consented prior to starting chemoradiotherapy.

Three patients commenced KD before starting chemoradiotherapy (n=3 MKD), seven during chemoradiotherapy (n=5 MCT KD, n=2 MKD; median delay 5 days (3-16 days); delay at patients request due to oncology appointments) and no patients commenced diet after completing chemoradiotherapy.

Dietary compliance

Patients who retained within the trial provided food diaries (MCT KD n=3; MKD n=1). The MCT KD group over consumed LCT fat (up to 65% of total energy intake; target 45%), whilst under consuming MCT fat (25% of total energy intake; target 30%) and under consuming carbohydrate (down to 5% total energy intake; target 10%). Reasons for non-compliance with MCT supplements were reported as excessive volume (n=3). Only one patient completed the trial in the MKD group, this patient was compliant with dietary targets for the duration.

Dietary adjustments required to achieve ketosis

Four patients required dietary adjustments from baseline calculations to improve ketosis. These changes took place during the first six weeks following dietary initiation and were tailed to the patients’ individual needs. All four patients were in the MCT KD group.

Dietetic time required for dietary interventions

The time associated with clinic visits and non-clinical activities during the first three months for MCT KD (n=3) was 6.9 hours (S.D ±0.2 hours) and 6.5 hours (S.D. ±1 hour) respectively. For MKD clinical time equated to 7.5 hours and 6.6 hours non-clinical (n=1). For a 12 month intervention a total of 10.1 hours clinical time and 10.8 (S.D. ±1.1 hours) non clinical time was required for MCT KD and 10.5 hours clinically and 10.1 hours non-clinical for MKD (details of activities undertaken can be found in the protocol (26)).

1. *KEATING study secondary outcomes: Impact of the trial on patients’ health*

Gastrointestinal side effects

During the first six weeks, the MCT KD group reported four incidences of gastrointestinal side effects and the MKD group two incidences. For the MCT KD group these were diarrhoea (n=1, CTCAE grade 1), nausea (n=1, CTCAE grade 1), vomiting (n=1, CTCAE grade 2), dyspepsia (n=1, CTCAE grade 1) and for the MKD group these were vomiting (n=1, CTCAE grade 1) and a dry mouth (n=1 MKD, CTCAE grade 1).

At month six, one patient (MCT KD) experienced dyspepsia (CTCAE grade 1), constipation (CTCAE grade 1) and diarrhoea (CTCAE grade 1) which led to their withdrawal from the trial. Another patient (MKD) also reported constipation (CTCAE grade 1).

No further gastrointestinal side effects were noted throughout the course of the trial.

Changes to biomarkers

Four adverse events were noted due to deranged biochemical markers; hypokalaemia (n=2, CTCAE grade 1), hypernatremia (n=1, CTCAE grade 1) and hypocalcaemia (n=1, not classified as adjusted calcium >2mmol/L).

Repeat cholesterol data was available for two patients in MCT KD group and one patient in MKD group. Total cholesterol to HDL ratio reduced over 12 months in both groups (MCT KD baseline ratio 4 [2-5], month 12 ratio 3.5 [3-4], n=2; MKD baseline 4, month 12 ratio 2, n=1).

Anthropometry changes

Repeat anthropometric measures were available for three patients in the MCT KD group and one patient in MKD group (see supplementary table B). For the MCT group (n=3), changes were clinically insignificant, with centile ranges being maintained for all measures. For the one patient following MKD, changes were clinically significant, with BMI reducing from obese to overweight, MAMC reducing from greater than 95^th^ centile to 15^th^ to 25^th^ centile range, waist circumference reducing from high risk to healthy and fat mass reducing from greater than 95^th^ centile to 50^th^ to 75^th^ centile range.

| **Supplementary table B: Changes in anthropometric measures over the duration of the trial** | | | | |
| --- | --- | --- | --- | --- |
| **Diet** | **Measure** | **Baseline** | **3 month review** | **12 month review** |
| MCT KD | Weight (kg) | 88.5  (±11.3) | 84.6  (±9.6) | 82.3  (±1.3) |
|  | BMI (kg/m2) | 29.1  (25.1-33.3) | 27.3  (25-31.9) | 27.2  (27.1-27.2) |
|  | MAMC (cm) | 29.5  (27.1-30.6) | 29  (27.1-29.1) | 26.6  (24.6-28.6) |
|  | Left HGS (kg) | 37  (32.8-47.6) | 32  (30.9-44.9) | 38.6  (28.2-49) |
|  | Right HGS (kg) | 35.9  (34.6-41.8) | 32.2  (31.7-40.4) | 27.9  (25.9-29.9) |
|  | Waist circumference (cm) | 97  (88-116) | 92  (88-112.5) | 94.8  (88.5-101) |
|  | Fat mass (%) | 28.3  (28.3-37.4) | 28.7  (22.6-35.3) | 28.4  (24.1-32.6) |
| MKD | Weight (kg) | 130.5 | 97.6 | 96.6 |
|  | BMI (kg/m2) | 35.8 | 31.9 | 28.9 |
|  | MAMC (cm) | 33.7 | 29 | 28.9 |
|  | Left HGS (kg) | 43.6 | 30.9 | 48.1 |
|  | Right HGS (kg) | 52.6 | 32.2 | 52.4 |
|  | Waist circumference (cm) | 124 | 112.5 | 93 |
|  | Fat mass (%) | 35.2 | 35.3 | 23 |

Abbreviations: BMI = body mass index; HGS = hand grip strength; MAMC = mid arm muscle circumference; MCT KD = medium chain triglyceride diet; MKD = modified ketogenic diet. NB: Baseline data MCT KD n=3, MKD n=1; three month data MCT KD n=3, MKD n=1; 12 month data MCT KD n=2, MKD n=1. Data presented as median (range).

Defining pilot success

There were five pre-defined criteria for pilot success. Table C describes the success criteria along with proposed amendments. A phase III KD trial for patients with GBM may be feasible if proposed amendments are implemented, but an internal pilot phase would be recommended.

| **Table C: Pilot success and proposed amendments** | | | |
| --- | --- | --- | --- |
| **Outcome** | **Pre-defined success criteria** | **Achievement** | **Amendments** |
| **Dietary retention** | Dietary retention rate of ≥75% (n=9) at three months | 33% (n=4) dietary retention at three months | Shorter intervention period of six weeks to be completed whilst undergoing chemoradiotherapy.  Use of an embedded qualitative study to explore patient experience and decision-making. |
| **Recruitment** | Recruitment rate of ≥75% of target (n=9) achieved within the 12 month recruitment period | 100% of recruitment target (n=12) within 12 months | NA |
| **Enrolment of patients** | ≥75% of patients commenced KD prior to chemoradiotherapy | 30% enrolled prior to chemoradiotherapy | KEATING protocol amended to allow patients to commence KD during or post treatment. Following which all patients enrolled prior to or within 16 days of treatment commencing.  Joint clinics with oncologist to reduce conflicting appointments. |
| **Food acceptability** | Diet acceptable to ≥75% of patients at three months | <50% due to withdrawals | Six week KD intervention.  Employ a validated questionnaire, if available. |
| **Completeness of data** | ≥75% of the proposed data collection completed for each end point | >75% completion rate for all outcomes, with the exception of ketone levels and dietary compliance at month 12 | Further PPI to investigate if digital solutions for the completion and return of food and ketone diaries would be acceptable to patients. |

Key: Achieved success criteria; Did not achieve success criteria.
