# Supplementary material for: Ketogenic diets as an adjuvant therapy for glioblastoma (KEATING): a randomized, mixed methods, feasibility study
Source: J Neurooncol. 2020 Feb 8;147(1):213–27. doi: 10.1007/s11060-020-03417-8 (PMC7076054; doi:10.1007/s11060-020-03417-8)
Supplement: Supplementary file 4 — Supplementary file4 (DOCX 114 kb) [file 11060_2020_3417_MOESM4_ESM.docx]

**Online resource 4: Qualitative study themes**

Patients’ accounts of their decision-making are presented in the following analysis according to the outcome of their decision about KEATING: i) continued participation; ii) early withdrawal; iii) delayed withdrawal; and iv) declined participation.

1. *Continued participation*

The three patients who continued to participate in KEATING beyond the three months recalled their decision to be instantaneous: *“I jumped in, you know, took the opportunity with both hands … it was a no brainer”* (T44). They emphasized that participating in the trial provided the opportunity for them to *“take control”* and *“fight for their life”* (T44).

After making their initial decisions, the three patients in this group reported seeking approval from their family: *“it was a case of speaking to my family and getting their support to make sure that they were on board with what I was going to do, my family gave me the thumbs [up]”* (T44). Caregivers of these patients who continued in KEATING supported the patients’ decision: *“[we would do] anything that works, to be honest, we’d give anything a go”* (T45/R).

Patients in this group also spoke of making an active decision to continue to participate on a regular basis, with all three making reference to the influence of ‘positive stories’ from long term ketogenic-glioblastoma survivors: *“there's lot of good results of people having positive responses to it [ketogenic diet]… the one story was the guy who had a, erm had the same tumor, he’s on this [ketogenic diet], his [tumor] reduced, what's not to want to go for that?”* (T45).

Patients explained their motivation for following the diet each day through *“long term goals”* (T44) and *“clear scans”* (T44), with ketones providing *“a quick confidence check and every now and again”* (T45) and offering ‘fulfilment’. Caregivers were supportive and emphasized the diet to be “*a new normal for us”* (T45/R).

1. *Early withdrawal*

Two patients consented to KEATING but withdrew early after being randomized and receiving dietary education, but before commencing the diet. Consistent with those who continued in KEATING, the two patients who withdrew early described their initial decision to participate as being *“more of a gut decision”* (T52) or based on *“gut instinct”* (T27), because *“[I] don’t want to die so I’ll try anything at the moment”* (T52).

It was not until later following the dietary education and after discussing their decision with a relative that they decided to withdraw, explaining that, on reflection, they were *“stressing myself out”* (T27) over continuing and had started to feel “*it was just too much, I was just trying to take too much on”* (T52). Both patients explained they felt that they would be reliant on the relative to assist them with the diet and monitoring during a ‘busy’ time, and this added to their ‘worry’ about participating in the trial.

1. *Delayed withdrawal*

Two patients withdrew after consenting to KEATING and following the diet for approximately six weeks, which we defined as delayed withdrawal. These patients also recalled their initial decision about participating in the trial to be instantaneous and different to how they usually made decisions: “*I thought about it but not as long as I would normally, I sort of made a decision on that day that I was going to have a go at it”* (T47).

These two patients also spoke of discussing their decision to participate with their relative *“we had a discussion together as to whether or not we felt it was the right thing for me to do… [relative] just supported me with it, he felt that I should be giving it a go as well”* (T47). Like the patients, caregivers also described their decision as instantaneous, “*I’d take anything with open arms because anything that would help cure [the tumor], you know… I’d jump at it”* (T47/R), with caregivers attributing a kind of selfishness to their motives: “*I wanted her to have a go… I suppose it’s a bit selfish really but you know you, there’s a selfish element in it because you want her to be here sort of thing”* (T39/R).

However, as time progressed initial dietary preferences could change with experience. These patients described feeling ‘worried’ about what they could eat: *“I was worrying, I was waking up, I was literally waking up… and that’s all I could think about: ‘Oh I've got to get my fats intake today’. And it was pulling me down”* (T39). Patients and caregivers recalled taking ketone measurements and finding these motivating when the readings were high but feeling ‘demoralized’ when the readings were low.

Patients and caregivers reported that following either KD would be difficult to *“live with that forever more”* (T47) and commented that three months following the diet was too long to sustain. This influenced their decision to withdraw from KEATING: “*it’s possibly too long on the diet*” (T47/R).

1. *Declined participation*

In comparison to those who consented, the three patients who declined KEATING described being considered and deliberate in their decisions, consistently describing a lack of perceived personal benefit from participation: *“the only thing I think about this study is erm what would benefit me”* (T35); *“if you were to say to me I guarantee that, I guarantee that, I guarantee you're going to be, better, you know, I might even do it”* (T55). They stated being ‘unconvinced’ by the research and that they were unlikely to experience any improvements in their quality of life: *“you get to around 70 years old and that’s where I am. So now every day I get up I want a quality day…and so having a complex regime around diet again it doesn’t appeal”* (T55). One viewed the trial as *“a waste of your life”* (T35), but reflected that they might have considered participating in KEATING for *“half of the time”* (T35).

All three who declined also recalled having discussed the trial with their relative, with two caregivers agreeing with the patients’ decision in relation to quality of life: *“well I think it’s something to be worthwhile but, erm, I was a bit concerned that it was a very restrictive diet for my wife to take at this stage really”* (T35/R). Following this discussion with their relative, these two patients reported feeling satisfied with their decision to decline. For the remaining patient, their decision to decline was not in agreement with their relative’s opinion, and this patient described weighing up the ‘pros and cons’ of the trial continuously and feeling uncertain over their decision.

Supplementary figure D offers an overview of patients’ decision-making patterns over the course of the trial.

Supplementary figure D: Qualitative pictorial of decision-making patterns

**
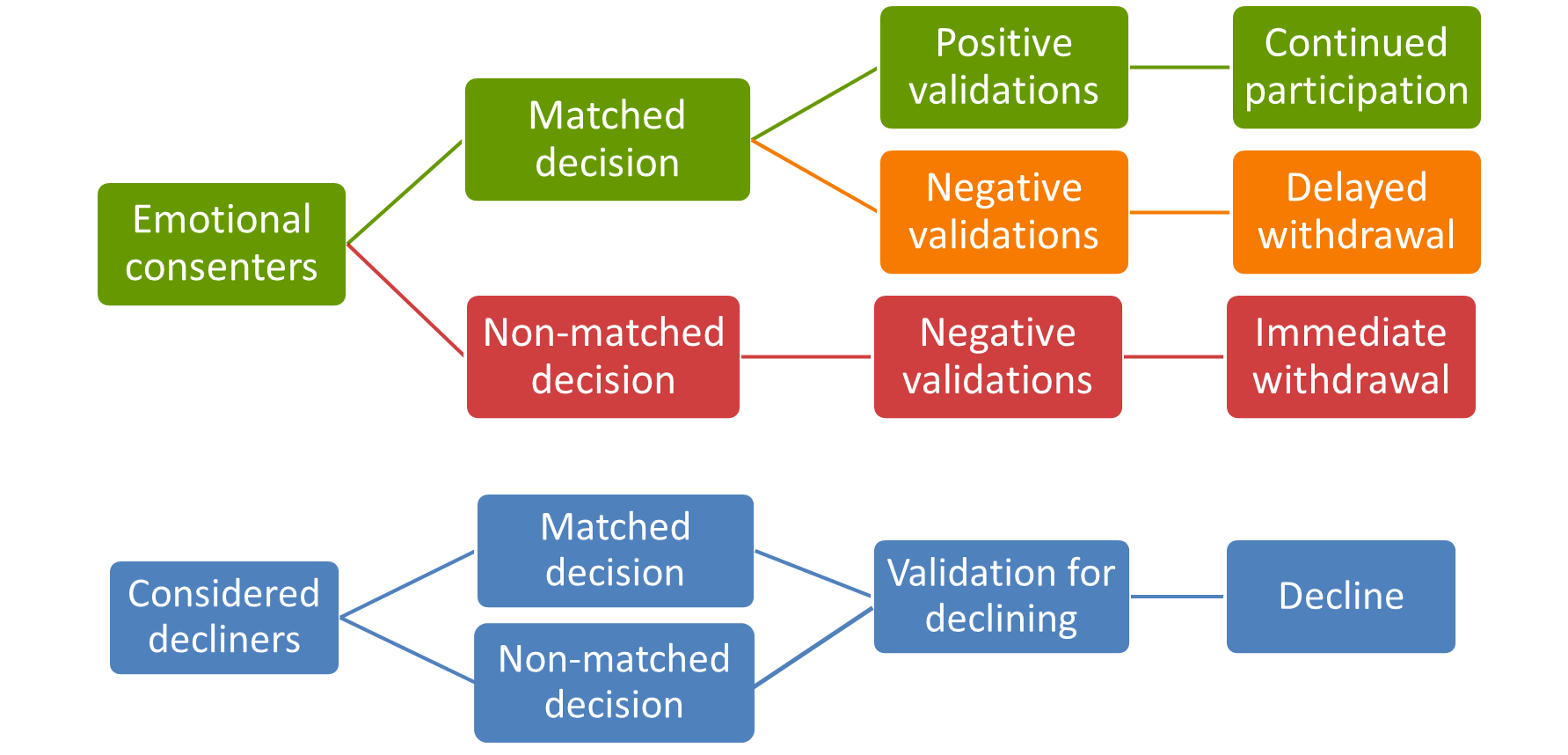
**

Key: Emotional consenters = patients who consented to participate in KEATING, intuitively; Considered decliners = patients who declined to participate in KEATING who were considered and deliberative in their decision-making. Matched decision = the decision of the patient is supported by their caregiver; Non-matched decision = decision of the patient is opposing to that of the caregiver; Positive validations = positive experiences or encounters which positively influence the patients’ decision to continue participating in the trial; Negative validations = negative experiences or encounters which negatively influence the patients’ decision to continue participating in the trial; Continued participation = continued with the intervention beyond three months; Early withdrawal = withdrew from the trial following consent and randomization, but prior to commencing a KD; Delayed withdrawal = withdrew from the trial after commencing KD but before the primary end point of three months; Declined = declined to participate in KEATING.
